# Erosion and deposition vulnerability of small (<5,000 km^2^) tropical islands

**DOI:** 10.1371/journal.pone.0253080

**Published:** 2021-09-16

**Authors:** Trevor N. Browning, Derek E. Sawyer

**Affiliations:** School of Earth Sciences, The Ohio State University, Columbus, Ohio, United States of America; Duy Tan University, VIET NAM

## Abstract

The tropics are naturally vulnerable to watershed erosion. This region is rapidly growing (projected to be 50% of the global population by 2050) which exacerbates erosional issues by the subsequent land use change. The issue is particularly of interest on the many (~45,000) small tropical (<5,000 km^2^) islands, and their >115M residents, where ecotourism and sediment intolerant ecosystems such as coral reefs are the main driver of their economies. However, vulnerability to erosion and deposition is poorly quantified in these regions due to the misclassification or exclusion of small islands in coarse global analyses. We use the only vulnerability assessment method that connects watershed erosion and coastal deposition to compare locally sourced, high-resolution datasets (5 x 5 m) to satellite-collected, remotely sensed low-resolution datasets (463 x 463 m). We find that on the island scale (~52 km^2^) the difference in vulnerability calculated by the two methods is minor. On the watershed scale however, low-resolution datasets fail to accurately demonstrate watershed and coastal deposition vulnerability when compared to high-resolution analysis. Specifically, we find that anthropogenic development (roads and buildings) is poorly constrained at a global scale. Structures and roads are difficult to identify in heavily forested regions using satellite algorithms and the rapid, ongoing rate of development aggravates the issue. We recommend that end-users of this method obtain locally sourced anthropogenic development datasets for the best results while using low resolution datasets for the other variables. Fortunately, anthropogenic development data can be easily collected using community-based research or identified using satellite imagery by any level of user. Using high-resolution results, we identify a development trend across St. John and regions that are both high risk and possible targets for future development. Previously published modeled and measured sedimentation rates demonstrate the method is accurate when using low-resolution or high-resolution data but, anthropogenic development, watershed slope, and earthquake probability datasets should be of the highest resolution depending on the region specified.

## Introduction

The tropics (±23.5° latitude) are prone to high erosion rates due to their consistently warm climate and prevalent rainfall both seasonally (higher latitudes) and year-round (near the equator) [1]. Many tropical areas are also on active tectonic settings that are steep and mountainous (e.g., Caribbean, Southeast Asia) contributing to high erosion rates [2,3]. Watersheds in these areas, especially on small islands, can have short sediment transport pathways to the coast [4] and brief watershed soil residence times, expediting delivery of watershed sediments to the coastal zone, as a result of consistent rainfall [5,6]. In addition, extreme events, such as monsoons, hurricanes, and earthquake-induced landslides, are common in the tropics and enhance erosion and infrastructure destruction [7]. With climate change expected to increase rainfall variability and extreme events such as hurricanes [8,9], higher erosion rates are expected. High population growth and the associated projected cropland expansion is further expected to exacerbate and increase terrestrial erosion rates [10].

There are ~45,000 islands >0.5 km^2^ within the tropics; most are small (only 78 are >1,000 km^2^) and, as such, are misclassified or ignored by global datasets and analyses [11,12]. The low resolution of global datasets, which is often on the kilometer scale or greater, means the vast majority of small (<1,000 km^2^) and medium-sized islands (1,000–5,000 km^2^) would be made up of less than a 1,000 grid cells or in some cases far less leading to misclassification of the overall data. Specifically, characteristics such as land cover, slope, and anthropogenic development (roads, structures) change rapidly on islands due to their small land area leading to misclassification or exclusion altogether. Tropical islands of this size contain a significant proportion of the global population, ~115.5M people or roughly 1/3^rd^ the current population of the United States [13].

Importantly, land-derived soil erosion is not a localized problem, but has cascading effects downstream on water quality, ecosystems, and coastal zones. The economies of small tropical islands (<1,000 km^2^) are commonly based on tourism driven by the natural ecosystems and landscapes. However, economically critical aquatic ecosystems [14–16] are at high risk to sedimentation (human and naturally induced) and the associated negative effects (mortality) that accompany it [17–19]. These include those that render important ecosystem services such as, calcareous algae (reef builders) [20], seagrasses (fish breeding grounds) [21], and coral reef communities (buffer the coast from waves/storms and increase biodiversity) [16,22]. Additionally, coastal seagrass communities are an important carbon capture and storage vehicle (~2x more efficient than tropical rainforests [23]). In freshwater ecosystems, sediment delivery and deposition degrades the quality of benthic habitats, and disrupts structural functions of freshwater ecosystems [24,25]. Fine-grained sediment and contaminants can cause microbial outbreaks and degrade water quality [26], alter water chemistry [27], and increase turbidity and suspended solid concentrations [28].

In response to rapid population growth (currently ~40%, projected 50% of the global population by 2040) and economic development, land use change is occurring regularly in the tropics, especially in the least developed countries (over 95% of which are in the tropics) [1,10,29]. Land use change is a main driver of enhanced erosion, primarily the conversion of forest to agriculture [10], which commonly occurs as a population and its food demand grows. In many cases, topsoil in agricultural and developed areas is anthropogenically replenished in a persistent cycle, which increases sediment loads to streams and coasts, halting bedrock weathering. Higher chemical bedrock weathering rates in the tropics consume a large component of global CO_2_ [2]. Despite the negative impacts associated with erosion and deposition, it is important to note that it is a natural process, which humans enhance in a positive feedback loop in multiple ways. For example, precipitation drives erosion, which climate change is projected to increase [9]. Greater amounts of sediment are then deposited, burying and killing downslope coastal ecosystems [17,18,30], which are carbon sinks [23]. Humans then add more sediment to the landscape, which disrupts weathering rates further exacerbating the carbon cycle [2]. In light of this land use change, understanding vulnerability to watershed erosion and coastal deposition is critical on small (<1,000 km^2^) developing tropical island nations which rely heavily on the resources or ecosystem services of their island (or islands) for their economy.

Assessments of vulnerability to erosion tend to focus either on the watersheds or the coastal zone, without connecting them, and typically are at the regional or watershed scale. For example, Coastal Vulnerability Indices have been used extensively in recent years to assess either coastal erosion [31–33] or watershed erosion [34–37]. Some focus on modeling potential watershed soil loss using the Revised Universal Soil Loss Equation (RUSLE) or other soil water erosion models, primarily at the watershed scale [38–40] with few at a global scale [41,42]. Recent studies in India have focused on integrating risk indices [43,44] for a more holistic approach by using multiple factors (physical, social, and geo-technical) to quantify risk to a region impacted by erosion.

Coastal Vulnerability Indices have not yet reached the global scale, focus on the coastline while ignoring watershed activities such as land use, and are generally at too large of a scale to be applicable for most small tropical islands. In contrast, RUSLE models focus on the potential soil loss in the watershed, but do not address sediment delivery to the coast or multiple types of land-use change. Despite recent improvements to RUSLE models [41,45] there are still accuracy issues in developing countries and remote small land area regions (Southeast Asia, the Caribbean, and Pacific islands) where high resolution datasets are scarce or non-existent. Thus, less accurate datasets are utilized to form the critical underpinning parameters of RUSLE such as the rainfall erosivity factor [46].

**Fig 1:**
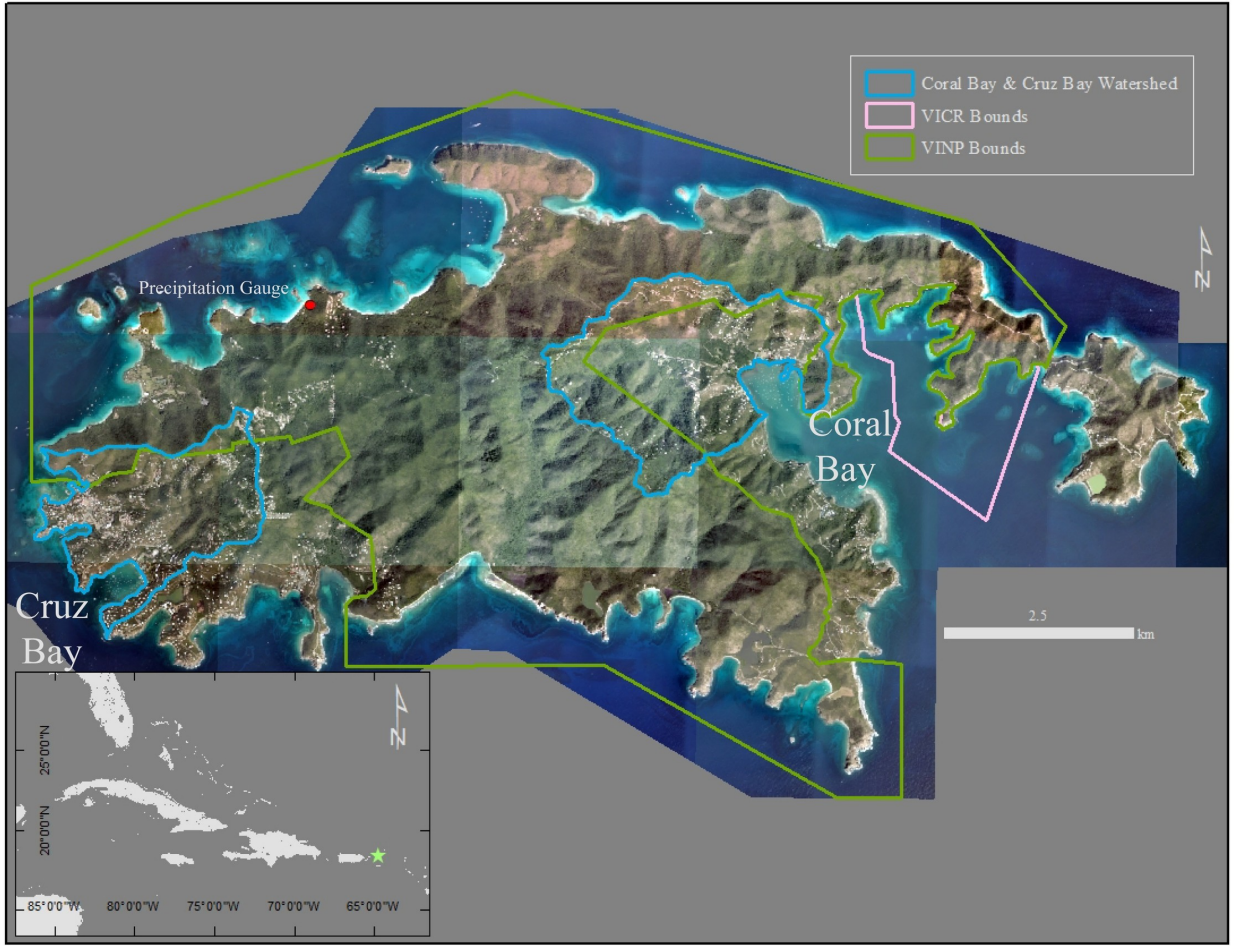
Satellite image of St. John, US Virgin Islands. Green star indicates the location of St. John on the inset map. The watersheds of Coral Bay and Cruz Bay are outlined in blue. The Virgin Islands National Park (green outline) encompasses the majority of St. John’s landmass while the Virgin Islands Coral Reef National Monument (pink outline) protects offshore of Coral Bay. Note the Virgin Islands Coral Reef National Monument covers a greater offshore area than is shown on this map. The location of the precipitation gauge used in the EVI-STJ is shown in Trunk Bay. Imagery Courtesy of **[53]**.

A new method developed by Browning and Sawyer [47] connects watershed erosion to coastal deposition across the tropics, termed the Erosion Vulnerability Index (EVI) and the coupled Erosion and Deposition Vulnerability Index (EDVI). This method focuses on land use change and open-source datasets in order to assess vulnerability to erosion and deposition across the entirety of the tropics. The EVI can be calculated for large areas while in order to calculate the EDVI and consider coastal deposition one must compute a watershed specific EVI. Our objective is to evaluate if low-resolution datasets (463 x 463 m) are accurate on small tropical islands (<1,000 km^2^). To do this, we use field-collected datasets [4,48,49] on St. John, in the US Virgin Islands. St. John is an ideal test site due to its size (50 km^2^), wealth of data on land [4,50] and coastal zone [48,49,51,52], and juxtaposition of heavily developed areas and undeveloped forests [4] (Fig 1).

### Geologic setting

St. John, like many other small tropical islands, is naturally susceptible to terrigenous (land-derived) sediment erosion due to its high-relief slopes, short pathways to the sea, and heavily weathered volcanic rocks [4,49,54,55]. In the early 1950’s the island was virtually undeveloped except a small community near Cruz Bay in western St. John (Fig 1). In 1956, over half of St. John was established as the Virgin Islands National Park (Fig 1). In the ensuing decades, development has increased substantially (40% permanent population increase since 1980 [56]) in support of increased tourism and part-time inhabitants [57]. Anthropogenic impacts over this period, primarily the construction of roads, have contributed to increased terrigenous sediment input into the marine environment [49,57–59]. In 2017, two separate Category 5 Hurricanes passed the island in a span of 2 weeks bringing excessive rainfall and damage to the infrastructure and ecosystems in the watershed and coastal zone [60]. Despite anthropogenically increased watershed erosion rates, the events deposited 100s of years sediment almost 100% of which was marine-sourced [48,60].

There are no perennial rivers or streams on St. John, only some spring-fed pools. Watersheds are drained by one or more gullies, locally known as “ghuts” [49,61]. Ghuts are only active during rainfall events that precipitate ≥12mm of rainfall per day, when erosional runoff is funneled down-slope to the coastal system [54]. The climate in the USVI is maritime tropical. A seasonal cycle of dry conditions in December−April is followed by a weak wet season in May−June, drier conditions in July−August, and a strong wet season in September−November [55]. The majority of precipitation occurs as short-duration heavy-rainfall events [55]. Precipitation is orographically controlled but on average the island receives around 1,200 mm/year and could range from ~700 mm to >2,000 mm.

Erosion issues on St. John have been studied with interest as development has increased and threatened these ecosystems following the incorporation of the Virgin Islands National Park [4,17,59,62–64].

St. John is representative to other small tropical islands vulnerable to erosion given its size (50 km^2^), mountainous topography, concentrated rainfall, and rapid development. Furthermore, the wealth of data on St. John allows for testing the performance of a global erosion vulnerability index [47]. The lack of perennial streams allows for a very accurate estimation of sediment flux and deposition rates around the island. This means that erosion and terrigenous deposition are driven by rainfall and thus the terrestrial signal is easy to identify, which aid in validating our qualitative results. We present results for all of St. John’s land area, including site-specific analysis of 2 watersheds using the low-resolution datasets from Browning and Sawyer [47] and new high-resolution field collected datasets.

## Methods

We assess vulnerability to erosion and deposition on St. John using locally or regionally collected high-resolution datasets input into the EVI-EDVI method described in Browning and Sawyer [47]. Step-by-step instructions on the analysis are shown in Appendix A. Importantly, the EVI method quantifies watershed erosion on the global scale but is unable to quantify deposition on the same scale. Thus, to quantify deposition, first an individual watershed must be selected for a watershed specific EVI calculation, then 3 coastal deposition variables are added to the EVI to determine a coupled erosion-deposition vulnerability index (EDVI) for that watershed and its coastal zone [47].

EVI is calculated from seven Risk Factors [47] as

$$EVI={(LC}^{2}*{AGMD}^{2}*L^{2}*{ST}^{2}*P^{2}*{WS}^{2}*{EQ}^{2})/7$$ (1)

Where LC is Land Cover Type, AGMD is Agriculture, Grazing, Mining and Development, L is Bedrock Lithology, ST is Soil Thickness, P is Mean Precipitation Deviation, WS is Mean Watershed Slope, and EQ is Earthquake Intensity Probability. The form of Eq 1 is adapted from the NASA Coastal Vulnerability Index [65] developed for the coastal United States. We have modified the original term “P” in Eq 1, which was s Mean Annual Precipitation, but here we prefer Mean Precipitation Deviation. Mean Precipitation Deviation is designed to capture rainfall variability instead of total mean annual rainfall. We use local precipitation data from Trunk Bay in St. John collected by a tipping bucket rain gauge from 1984–2017 (Fig 1, Table 1). We calculate each individual year’s annual total subtracted from the 34-year average to determine the deviation, either positive (flood year) or negative (drought year) (there were no years without change). Drought years and flood years were averaged and quantified as mm below, or above, the 34-year mean. The difference between these two values is the Mean Precipitation Deviation, essentially the difference between an average drought year and an average flood year.

**Table 1:** All variables used in the EVI-STJ & EDVI-STJ with their resolutions and sources.

Dataset Name	Data Type	Measured by	Acquired Year	Dataset Type	Resolution (meters)	Data Originator
Land Cover Type	Land Cover Type	Imagery and Algorithm	2012	Raster Grid	1	[66]
Mean Watershed Slope	Digital Terrain Model	LiDAR	2013	Raster Grid	5	[67]
Soil Thickness	Estimated Topsoil Thicknesses	Interpolated from Field Samples	2019	Raster Grid	1	[68]
Lithology	Lithology	Local Field Surveys	N/A	Vector Polygons	N/A	Browning, T.N. Digitized [69]
Mean Precipitation Deviation	Precipitation	Tipping Bucket Rain Gauge	1986–2017	Vector Points	N/A	Rafe Boulon
Agriculture	Land Cover Type & Satellite Imagery	Imagery and Algorithm	Present	Raster Grid	1	Assessed by Browning, T.N. using images from Google, Digital Globe and [66]
Grazing	N/A	Satellite Imagery	Present	N/A	N/A	Assessed by Browning, T.N. and CBCC using images from Google, Digital Globe
Mining	N/A	Satellite Imagery	Present	N/A	N/A	Assessed by Browning, T.N. and CBCC using images from Google, Digital Globe
Development	User Created	Satellite Imagery & Census Data	2018–2019	Vector Points	N/A	Assessed by Browning, T.N. using images from Google, Digital Globe and data from CBCC and [56,70]
Earthquake Intensity Probability	Peak Ground Acceleration	Measured and Modeled Data	2003–2053	Raster Grid	1500	[71]
Mean Marine Coastal Slope	Digital Elevation Model	LiDAR and Bathymetry Measurements	2011 & 2017	Raster Grid	3	[48,72]
Fluvial Sediment Input	Sediment Delivery Estimates	Field Measurements and Modeling	2017	Vector Polygons	N/A	[48,73]
Coastal Protection	Aerial Photos	Satellite	2015–2018	TIFF Image	N/A	Assessed by Browning, T.N. using images from Google, Digital Globe

Several of the high-resolution datasets in the EVI-STJ were created or modified using satellite imagery to enhance the accuracy of the original data or digitized from previous publications to get the most accurate, site-specific data possible (Table 1). Specifically, Lithology was digitized from Alminas, Foord [69] representing the most recent comprehensive lithologic survey map completed on St. John (Table 1). Multiple datasets misclassified the many salt ponds that line the coast of St. John (small ponds fed by ocean water but disconnected from the coast by a small strip of sand). We omitted these from our watershed EVI-STJ because, as basins, they will tend to intercept eroded watershed sediments. The individual components of the Agriculture, Grazing, Mining, and Development (AGMD) datasets were compiled and modified from existing datasets created by the Coral Bay Community Council (CBCC), US Census Data [56], and satellite imagery (Table 1). Mining and Grazing activities have either never occurred on St. John or ceased long ago (Personal Communication, CBCC), this was confirmed via current satellite imagery (Table 1). Only one small agricultural plot exists on the island (Personal Communication, CBCC), which was verified using current satellite imagery (Table 1). The Development component of AGMD is made up of houses, roads, and paved areas. A CBCC housing shapefile was updated via satellite imagery from 2018 to reflect new construction. Roads were delineated using data from Browning, Sawyer [4] and the TIGER/Line 2017 shapefile for St. John from the US Census [70] (Table 1). This combination file was verified using 2018 satellite imagery to reflect current conditions (Table 1).

After generating the watershed-specific EVI-STJ from Eq 1, three additional variables designed to address the vulnerability to coastal deposition (Mean Coastal Marine Slope, Fluvial Sediment Input, and Coastal Protection) are added to the EVI to generate the EDVI-STJ. The values for the Mean Coastal Marine Slope variable in Cruz Bay come from NOAA’s 2011 bathymetry dataset [72] while values for that same variable in Coral Bay come from bathymetry data collected by Browning, Sawyer [48] in September of 2017 (Table 1). Due to the small size of the island and watershed, we calculated Mean Coastal Marine Slope by using a 3 km slope transect rather than 10 km in Browning and Sawyer [47]. We use modeled sediment delivery rates to estimate the Fluvial Sediment Input variable. Although this is the only modeled product used, maximum sediment delivery estimates established for all Coral Bay watersheds during 2017 (largest flood year in our 34 year dataset) would still be classified on the lowest end of the Very Low Risk Factor [48] (Table 1). Coastal Protection is identified using satellite imagery. EDVI Vulnerability Classes are then established using Table 2 [47].

**Table 2:** Risk Factor (RF) distribution in Risk Categories for the tropical Erosion Vulnerability Index (EVI) and Vulnerability Classes for watershed EVI and Erosion and Deposition Index (EDVI). These categories are also used for the EVI-STJ and EDVI-STJ. Table modified from Browning and Sawyer, 2021 [47].

**Risk Factors**					
AGMD	2	3	3	3	5
Bedrock Lithology	2	2	3	3	5
Land Cover Type	1	1	3	4	5
Mean Annual Precipitation	2	1	2	4	5
Mean Watershed Slope	1	3	2	4	5
Earthquake Intensity Probability	2	2	2	4	5
Soil Thickness	1	2	3	2	5
**Risk Categories**	Very Low (1)	Low (2)	Medium (3)	High (4)	Very High (5)
Risk Category Ranges	0.14–36.57	36.57–740.57	740.57–59,986.29	59,986.29–3,033,380.57	3,033,380.57–871,930,803.57
**Vulnerability Classes**	Very Low	Low	Medium	High	Very High
Upper Break Risk Category Distribution (percentage of grid cells)	51% 2 49% 1	51% 3 49% 2	50% 4 30% 3 20% 2	75% 4 15% 5 10% 3	100% 5
Vulnerability Class Range	100–151	151–251	251–330	330–405	405–500

After compiling the high-resolution datasets for the EVI-STJ and EDVI-STJ, we compare this to the results of the low-resolution [47] EVI & EDVI to quantify the extent that low-resolution datasets misclassify risk on small tropical islands (Table 3). We demonstrate the differences on the island scale as well as the watershed scale in two different developed basins on St. John.

**Table 3:** Summary results and comparison of applying the high-resolution EVI/EDVI approach described in this study relative to the global EVI/EDVI approach (Browning and Sawyer, 2021 [47]). Erosion Vulnerability Index (EVI). Erosion and Development Vulnerability Index (EDVI).

Site	EVI	EDVI	Dominant EVI Risk Category	Dominant EDVI Risk Category
Cruz Bay (Browning and Sawyer, 2021) [47]	Low	Medium	Low (59%)	Medium (36%)
Cruz Bay (This Study)	High	High	Medium (54%)	Medium (44%)
Coral Bay (Browning and Sawyer, 2021) [47]	Medium	Medium	Medium (89%)	Medium (45%)
Coral Bay (This Study)	High	Very High	Medium (74%)	Very High (71%)

## Results

### Classified variables (Risk Factors) for the high-resolution EVI-STJ

**Fig 2:**
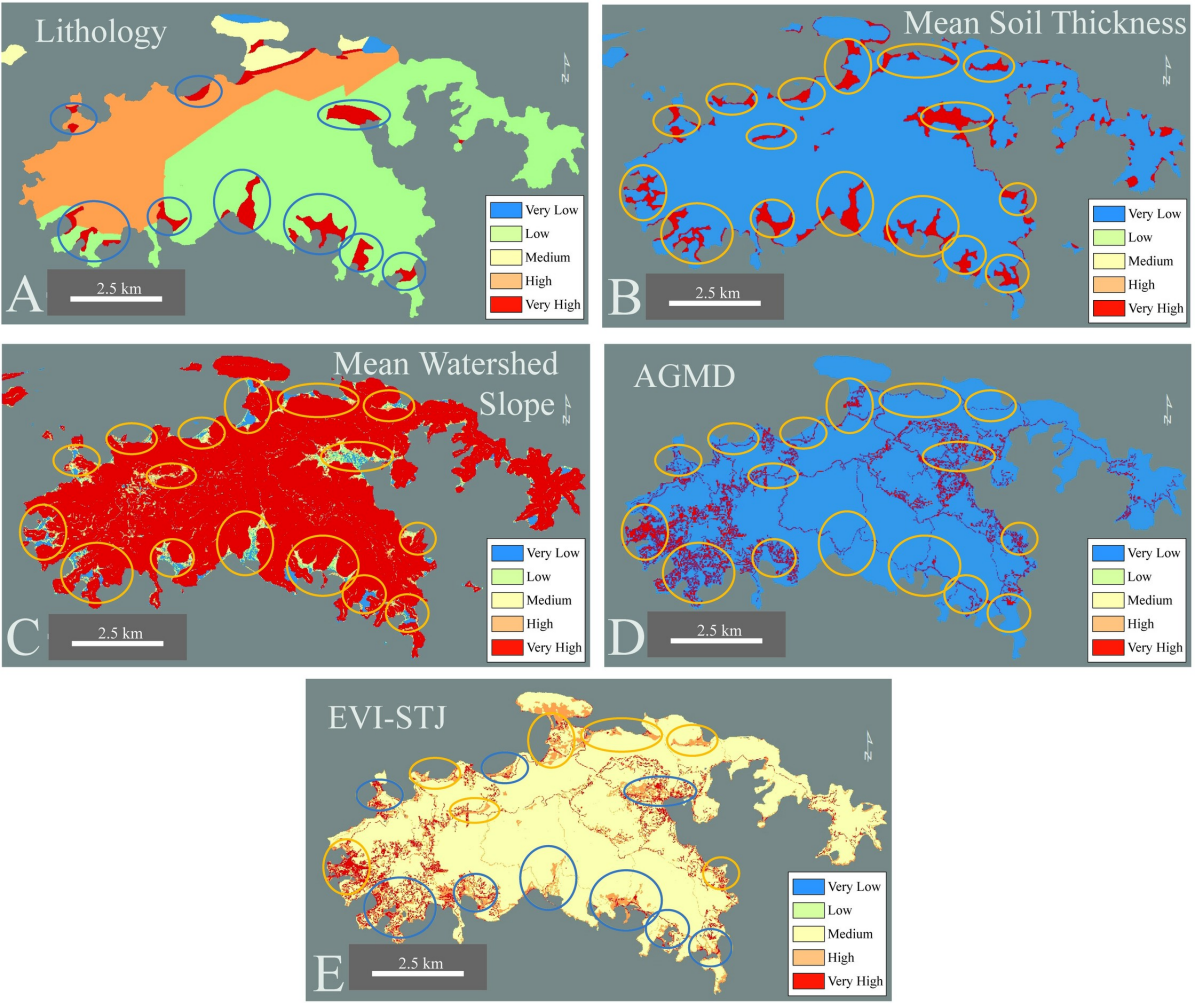
Comparison of Risk Factors for Lithology (A), Mean Soil Thickness (B), Mean Watershed Slope (C), AGMD (D), and the EVI-STJ (E). Very High Risk Factors for Lithology and Mean Soil Thickness Correlate with Low Risk Factors for Mean Watershed Slope. Low-lying areas or basins collect alluvium and greater soil thicknesses. Due to this, these areas have higher Risk Categories for the EVI-STJ. Much of the AGMD is concentrated in these areas. Lower slope areas are easier to build on and thicker soil mantles allow for easier construction. Thus, these circled regions (if currently lacking AGMD) could be regions that are targeted for development in the future.

**Fig 3:**
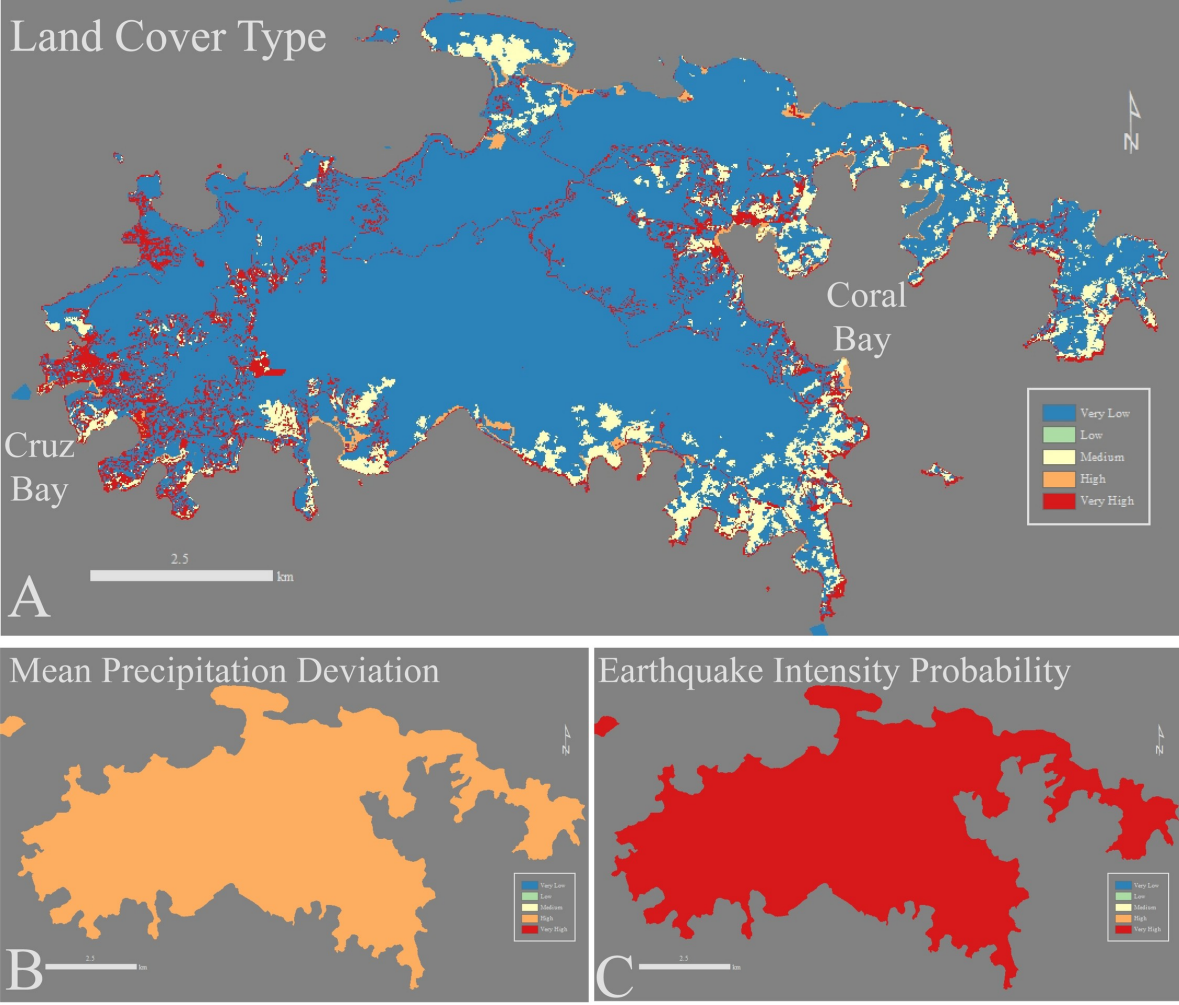
Land Cover Type (A), Mean Precipitation Deviation (B), and Earthquake Intensity Probability (C) used in the EVI-STJ on St. John, USVI. Land Cover Type is similar to AGMD (Fig 4) but misses many of the roads in the southern portion of the island. Most of the island is forested due to the Virgin Islands National Park.

**Fig 4:**
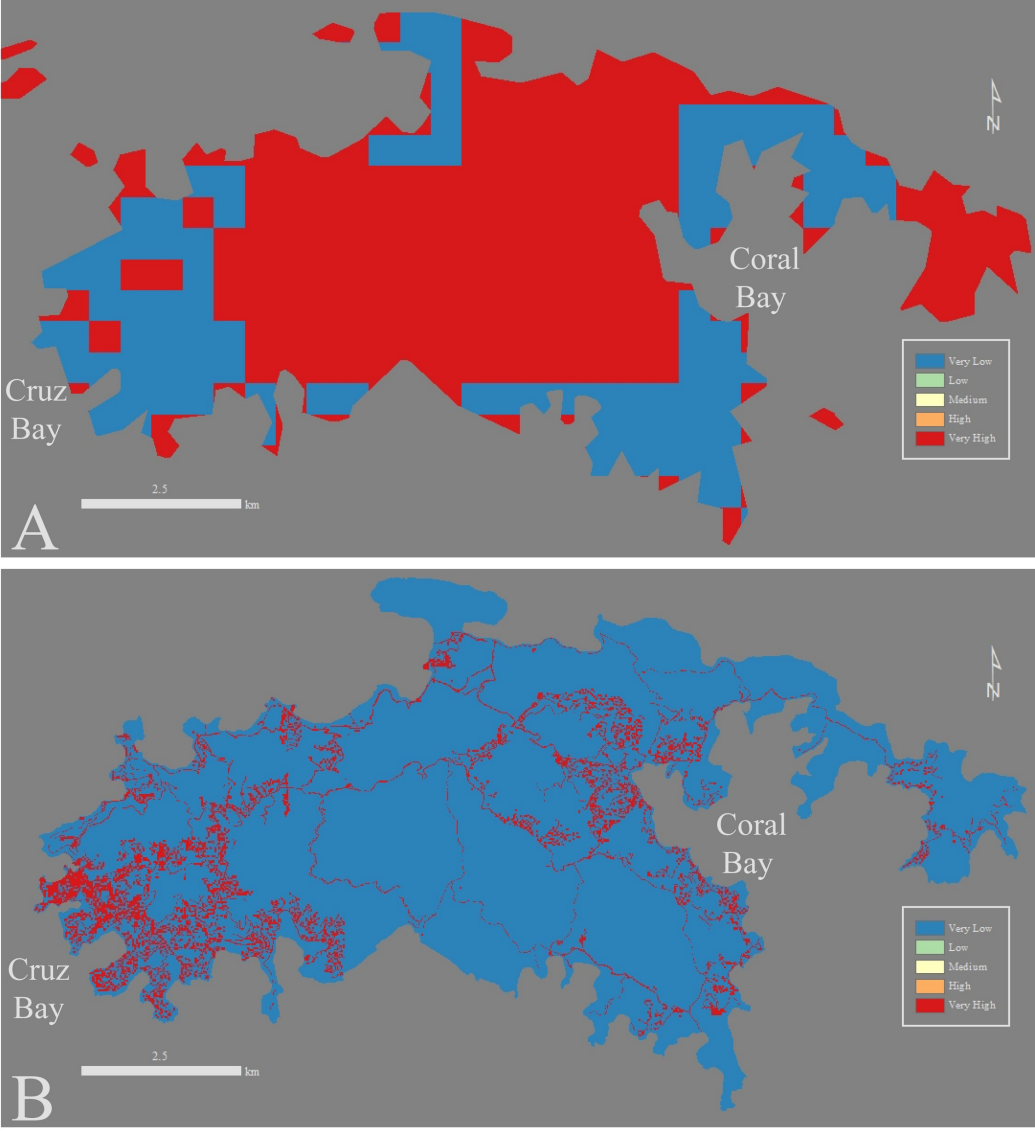
Agriculture, Grazing, Mining, and Development (AGMD) comparison between the global EVI (A) and EVI-STJ (B). The resolution differs drastically, when using higher resolution data the overall Risk Factors decrease and invert the pattern shown in Browning and Sawyer [47] EVI.

Here we present each Risk Factor over the entirety of St. John using new high-resolution data (5 meter x 5 meter grid cells, Figs 2 and 3). The resolution of 5 m x 5 m grid cells was dictated by the underlying resolution of the datasets used. Agriculture, Grazing, Mining, and Development (AGMD) is a binary variable and thus is only displayed as Very Low or Very High Risk Factor. Very High Risk Factor grid cells for AGMD are concentrated near the two towns, one on the west coast of the island (Cruz Bay) and the other in the eastern coast region (Coral Bay, Figs 1 and 4). Most of St. John is forested (part of the Virgin Islands National Park, Fig 1) and thus primarily Very Low Risk Factor values for Land Cover Type (Fig 3 Part A). Very High and Medium Risk Factors for Land Cover Type align with deforested areas near the coast or in developed areas similar to AGMD (Figs 3 and 4). The eastern half of the island’s lithology is igneous leading to a Low Risk Factor classification while the western half is sedimentary resulting in High Risk Factors (Fig 2 Part A). Isolated areas of Very High Risk Factors are associated with Holocene-aged alluvium in lowland areas near the coast (Fig 2 Part A).

Mean Watershed Slope on St. John is very steep (> 45° in many areas) leading to Very High Risk Factors over most of island (Fig 2 Part C). Lower Risk Factors exist near the coast as the slope decreases in lowland areas. Soil thicknesses on the majority of the island are thin leading to dominant coverage of Very Low Risk Factors of Mean Soil Thickness (Fig 2 Part B). The few Very High Risk Factors for Mean Soil Thickness are concentrated in lowland areas that are able to trap more sediments leading to higher soil thicknesses (Fig 2 Part B). On an annual scale, Mean Precipitation Deviation variable is unlikely to change significantly over this small an area (~15 km x 8 km). Thus, at 445 mm it is shown as High Risk Factor for the entire island due to the strong flood and drought seasons they have cyclically (Fig 3 Part B). Earthquake Intensity Probability has a Very High Risk Factor due to the large amount of faults and the generally active tectonic zone that surrounds the Greater Antilles (Fig 3 Part C) [71].

### Island-wide high-resolution EVI-STJ

**Fig 5:**
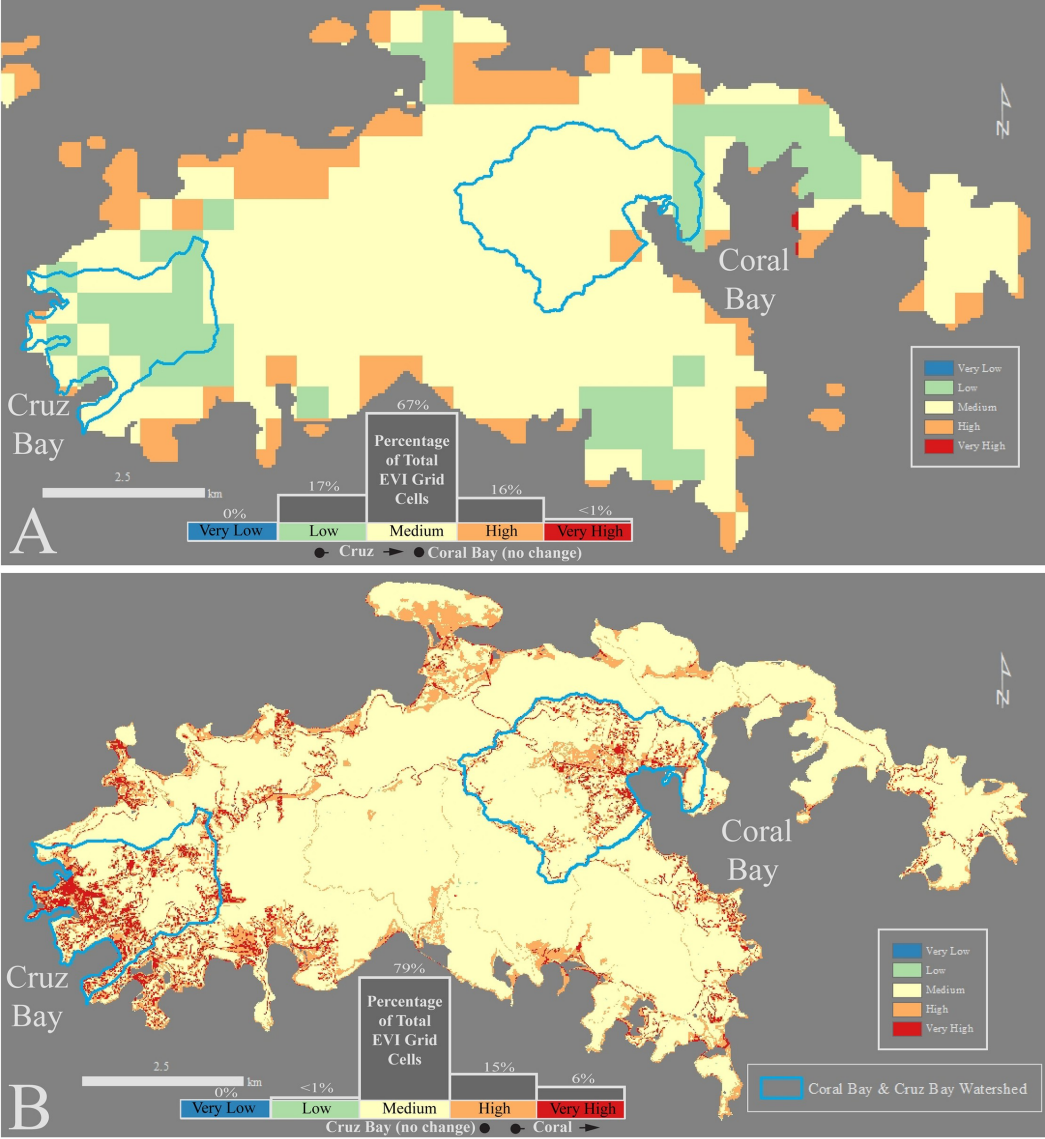
Improved resolution between the global EVI (A) and the EVI-STJ (B). Stark differences in vulnerability are revealed, concentrated in the two major communities of Cruz Bay and Coral bay.

The EVI-STJ is composed of predominantly Medium Risk Factor grid cells (79%) focused on the central portion of the island (Fig 5). The remaining cells are composed of High and Very High Risk Factors (15% and 6% respectively) concentrated near the two settlements (Cruz Bay and Coral Bay) and along the coast (Fig 5). There are no Very Low Risk Factors cells and <0.01% Low Risk Category cells.

### Cruz Bay: High-resolution EVI-STJ and EDVI-STJ

The watershed EVI for Cruz Bay is predominately composed of Medium Risk Factor grid cells (54%) with even amounts of High and Very High Risk Factor cells (25% and 21% respectively) (Fig 5 and Table 3). Very High Risk Factor grid cells are concentrated near the greatest amount of development close to the coast (Figs 1 and 5). There are no Very Low Risk Factor grid cells and few (<0.1%) Low Risk Factor cells. Overall, the Cruz Bay watershed profiles in the High Risk Category (Table 3). Accounting for coastal deposition, the EDVI-STJ for Cruz Bay does not change the watershed EVI-STJ also profiling in the High Vulnerability Class (Table 3). This is due to the variability in 3 EDVI Risk Factors from Very Low (Fluvial Sediment Input), Medium (Coastal Protection), to Very High (Mean Coastal Marine Slope).

### Coral Bay: High resolution EVI-STJ and EDVI-STJ

The watershed EVI-STJ for Coral Bay is primarily made up of Medium Risk Factor grid cells (74%) with some High (18%) and Very High Risk Factor (8%) cells (Table 3). Similar to Cruz Bay the High and Very High Risk Factor cells are concentrated near anthropogenic development and in low slope areas where construction of roads and structures is easier. There are no Very Low Risk Factor grid cells and few Low Risk Factor cells (<0.1%). Overall, the watershed EVI-STJ in Coral Bay was classified in the High Risk Category (Table 3). When including coastal deposition in the EDVI-STJ the vulnerability increases up to the Very High Vulnerability Class (Table 3). This is driven by Very High Risk Factors for Mean Coastal Slope and Coastal Protection, despite a Very Low Risk Factor for Fluvial Sediment Input (Table 3).

## Discussion

### Low-resolution EVI variables misclassify risk compared to high-resolution EVI-STJ analysis

Overall, the low-resolution datasets used in a global EVI analysis [47] lead to misclassified risk on St. John with the difference more stark at the watershed scale. The misclassification is driven by the low resolution of the AGMD and Mean Watershed Slope datasets and the inaccuracy of the Earthquake Intensity Probability dataset [74] when compared to higher-resolution datasets (Table 3). The increased resolution used in the new EVI-STJ data (~5 m) over the global EVI resolution (463 m) is demonstrated by Fig 5.

#### Minor change in vulnerability at the island scale

Although the difference in resolution between the global EVI and the EVI-STJ is large, the difference in island-wide risk distribution is fairly subtle. Overall, the low-resolution global EVI on St. John consisted primarily of Medium Risk Factor cells (67%) with some Low and High Risk Factor cells (17% and 16% respectively), few Very High Risk Factor cells (<1%) and no Very Low Risk Factor cells (Fig 5). The high-resolution EVI-STJ increased the percentage of Medium Risk Factor cells (79%) and Very High Risk Factor cells (6%) while Low Risk Factor Cells decreased (< 1%) and High Risk Factor cells remained similar (15%) (Fig 5). Overall, the difference in risk was driven by the scarcity of Very Low and Low Risk Factors in the EVI-STJ.

#### Major difference in vulnerability at the watershed scale

While the changes in the overall EVI on St. John were minor, changes within some watersheds were much more evident. We examined this difference further in 2 of the most developed watersheds on St. John: Cruz Bay and Coral Bay (Fig 1). Cruz Bay watershed was originally classified in the Low Risk Category in the global EVI while the high-resolution EVI-STJ classified it two Risk Categories higher, in the High Risk Category (Table 3). In Cruz Bay, the original EVI yielded predominately Low (59%) and Medium Risk Factor grid cells (39%) with little High Risk Factor cells (1%) and no Very Low or Very High Risk Factor cells (Fig 5). Almost all Low Risk Factor grid cells are gone (<0.01%) replaced by High and Very High Risk Factor cells (25% and 21% respectively) (Fig 5, Table 3). This change is primarily driven by the severe misclassification of the AGMD variable, which we describe in detail in the Discussion Section under Recommendations (Fig 4).

The difference between the global EVI and the EVI-STJ was less drastic in Coral Bay. Coral Bay is naturally more vulnerable than Cruz Bay due to High and Very High Risk Factors for Lithology and Mean Soil Thickness, which Cruz Bay lacks (Fig 2). In the global EVI, Coral Bay was composed dominantly of Medium Risk Factor grid cells (89%) and small amounts of Low and High Risk Factor Cells (8% and 3% respectively) (Table 3). The increase in risk shown in the EVI-STJ in Coral Bay was driven by a large increase in High and Very High Risk Factor cells (18% and 8% respectively) (Table 3). Low Risk Factor cells in the EVI-STJ in Coral Bay disappeared (<0.1%) while Medium Risk Factor cells decreased (74%). These changes are very similar to what occurred in Cruz Bay driven primarily by AGMD misclassification.

Importantly, EVI analysis can identify watersheds more, or less, vulnerable to sediment erosion but does not inform what may happen in the downstream coastal system. It is important to evaluate this as some coastal settings may be more, or less, suitable to handle an increase in sediment load. After calculating the watershed-specific EVI, the Risk Factors for each of the three coastal deposition variables (Table 1) are added to yield the EDVI (Table 1). In Cruz Bay, the coastal Risk Factors for these EDVI variables were classified as Very High Risk Factor for Mean Coastal Marine Slope, Very Low Risk Factor for Fluvial Sediment Input, and Medium Risk Factor for Coastal Protection. In Coral Bay, the coastal variables were classified as Very Low Mean Coastal Marine Slope, Very Low Risk Factor for Fluvial Sediment Input, and Very High Risk Factor for Coastal Protection.

For Cruz Bay, the Low Risk Category in the watershed EVI moved up to the Medium Vulnerability Class in the EDVI (Table 3). This was driven by a Very High Risk Factor for Mean Coastal Marine Slope (Table 3). In the high-resolution analysis, the risk in Cruz Bay did not increase. Since the watershed EVI-STJ was classified in the High Risk Category, the incorporation of more Medium and Very Low Risk Factors (for Coastal Protection and Fluvial Sediment Input) slightly decreased the risk of the EDVI-STJ (from a Risk Category value of 367 to a Vulnerability Class value of 333, Table 2) though did not change its ranking, remaining in the High Vulnerability Class for the EDVI-STJ (Table 3).

For Coral Bay, the risk does not change from the watershed specific EVI classification (Medium Risk Category) in the EDVI (also Medium Vulnerability Class) (Table 3). However, in the high-resolution analysis the risk increases from the High Risk Category in the watershed EVI-STJ to Very High Vulnerability Class in the EDVI-STJ (Table 3). This is driven by Very High Risk Factors for Mean Coastal Slope and Coastal Protection. These Risk Factors are the same in the Browning and Sawyer [47] analysis except Mean Marine Coastal Slope which changes drastically between the two analyses.

The disparities caused by the inaccuracy of the AGMD are especially apparent in Cruz Bay where the EVI analysis between the studies differs by two Risk Categories (Table 3). Satellite imagery and classification schemes used to determine Land Cover Type and AGMD in the global EVI [47] such as LandSAT and MODIS struggle to penetrate dense vegetation and thus can miss roads and houses, which is the primary type of AGMD on St. John. Using census data combined with many different years of satellite imagery and GPS surveys [4] the EVI-STJ is better informed. These new data invert the trend shown in [47] with Very High Risk Factors near the coast and towns (Cruz Bay and Coral Bay) leading to the drastic differences in risk classification between these two studies (Fig 4, Table 3).

### Identification of an anthropogenic development pattern

Development appears to be concentrated in areas with favorable building conditions (low slope, thick soils), which align with Very High and High Risk Factor EVI grid cells for multiple variables (Fig 2). Due to extremely steep slopes on St. John [4], little alluvium (Very High Risk Factor for Lithology) or thick soils (Very High Risk Factor for Mean Soil Thickness) exist on most of the island (Fig 2). However, in low slope areas alluvium and soil mantles can accumulate (Fig 2). These regions seem to have been targeted for development and generally feature clusters of homes and accompanying Very High Risk Factors for AGMD (Figs 1 & 2). Loose alluvium has higher vulnerability to erosion than bedrock and generally collects in basins or lower slope areas and thus thicker soils are found in these regions (Fig 2, Table 1). These areas with low slope (Low Risk Factor) but thick soil (Very High Risk Factor) are also good areas to build and establish infrastructure. Construction on steep slopes is more challenging than on flat ground, and thin soil thicknesses associated with those areas means that builders will have to drill through bedrock to establish solid foundations (Fig 2).

The clearing of land for infrastructure removes vegetation from the landscape, which destabilizes the soils that are unpaved, leading to enhanced erosion and higher peak watershed flows [58]. Paved areas, especially roads, preclude infiltration of water into the ground leading to increased peak flows, which lead to higher erosion and sediment delivery rates to the coast [58]. Fig 2 highlights this, where most of the circled zones have Very High or High Risk Factors for Lithology, Mean Watershed Slope, or Mean Soil Thickness. Many of the circled areas have Very High Risk Factors for AGMD meaning they have already been developed. Circled regions that have Very Low AGMD could be new potential areas of development, but risky options given the Very High Risk Factors for Mean Soil Thickness and Lithology.

### Validating vulnerability assessments of erosion and deposition in Coral Bay

The global EVI and EDVI analysis underestimated Coral Bay’s vulnerability by a Vulnerability Class but was more accurately assessed in the EVI-STJ & EDVI-STJ when validated against modeled watershed sediment yield estimates and calculated deposition rates [48,73]. The maximum sediment flux estimate for Coral Bay is 0.0004 Tg/year, which would be a Very Low Risk Factor for Fluvial Sediment Input [48]. The dataset used in the Browning and Sawyer [47] EVI [75] showed the entire St. John region as less than 1 Tg/year which is reasonable (or possibly even too high) considering one of the largest watersheds on the island delivers 0.0004 Tg/year [48].

Even though there is low fluvial input, Coral Bay is classified in the Very High Vulnerability Class in the EDVI-STJ due to Very High Risk Factors for two EDVI-STJ variables. This assessment is validated by the work done by Brooks, Larson [49] that demonstrate an increase in mass accumulation rates in coastal bay sediments following significant land use activity in the watershed. The anthropogenically altered mass accumulation rate of 0.15 g/cm^2^/yr in Coral Bay is not as large as may occur in other regions (thus the Very Low Risk Factor for Fluvial Sediment Input). However, this rate represents a vulnerability to this specific system due to Very High Risk Factors for Mean Marine Coastal Slope and Coastal Protection. The scientific community has documented this change and negative implications for decades, which is represented by the Very High Vulnerability Class EDVI-STJ ranking [17,62,64,76]. This is one instance where the EVI & EDVI method is validated by both modeled watershed data and measured sedimentation rates in the coastal zone. Due to consistent terrestrial input, resuspension, recirculation, and general mixing of coastal sediments, calculating coastal sedimentation rates can be very difficult. The system on St. John lacking perennial streams provides a more easily distinguishable sedimentological record in the coastal zone making it simpler to more accurately isolate than in most coastal sedimentation rates.

### Recommendations and future directions

By utilizing higher-resolution datasets the values of the Browning and Sawyer [47] EVI & EDVI were shown to change on the watershed scale in some cases demonstrating that the accuracy of the analysis will increase by using some high-resolution data. In both the Browning and Sawyer [47] EVI & EDVI and the EVI-STJ & EDVI-STJ analysis of Cruz Bay and Coral Bay the EDVI variables remain unchanged despite using higher resolution datasets. This implies that the resolution of these variables is not as critical as others in this region. The Coastal Protection variable remains unchanged because satellite imagery is used to obtain that Risk Factor in both the high- and low-resolution analyses. Fluvial Sediment Input does not change due to the island having no perennial rivers and thus no consistent sediment input [54,61]. Increasing the resolution of the bathymetry increases the accuracy of Mean Coastal Slope Risk Factor. In Cruz Bay and most other bays around St. John, this had little effect and did not change the assessed Risk Factor between studies. However, the unique geometry of Coral Bay caused issues with the dataset used to calculate Mean Marine Coastal Slope [77] in the Browning and Sawyer [47] analysis. Further analysis of the surrounding region using the [77] datasets confirms that this is an overlooked data anomaly due to the high terrestrial slopes completely surrounding a large coastal zone like Coral Bay and is likely to be rare throughout the tropics (Fig 1). Consulting a local nautical chart is suggested in order to confirm the slope given by the [77].

Unlike the EDVI variables, some watershed EVI variables should be higher resolution while others can remain at a lower resolution. Datasets like Mean Precipitation Deviation, Lithology, and Soil Thickness will improve the analysis but since they are slow to change and unlikely to vary over distances of ~500 meters, they are not critical for an accurate vulnerability assessment. Land Cover Type, though likely to change over ~500 meters, did not differ drastically enough between the Browning and Sawyer [47] analysis and the EVI-STJ to justify a more specific dataset.

We believe that high-resolution data for the Agriculture, Grazing, Mining, and Development (AGMD) variable are of most importance followed by Mean Watershed Slope and Earthquake Intensity Probability in certain situations. The difference between the Earthquake Intensity Probability dataset used in the global EVI and the EVI-STJ was significant (resulting in Low Risk Factor in the EVI and Very High Risk Factor in the EVI-STJ across the entire island). The analysis used for the EVI-STJ was done specifically for the Puerto Rico and the Virgin Islands and brings in regional data [71,78] used in the original. Mean Watershed Slope changes too drastically on St. John to rely on such a coarse dataset to inform the EVI. Thus, Earthquake Intensity Probability and Mean Watershed Slope should be of higher resolution if the region of interest is tectonically active or steeply sloped.

In general, the global EVI and EDVI underestimated the vulnerability of Cruz Bay and Coral Bay when compared to EVI & EDVI-STJ due to misclassification of the AGMD variable. The EVI overestimated the amount of Low Risk Factor cells in Coral Bay because it was unable to accurately identify important land use change activities such as roads and structures (Fig 4). Human development and land use change (AGMD variable) is difficult to quantify via satellite on a smaller scale in a dense tropical forest setting such as St. John. Unfortunately, this is a major issue because of the large impact that roads have on watershed erosion and sediment delivery to the coast [58]. We suggest, above all else, it is critical to identify roads, structures, agriculture, and mines in the landscape which can greatly alter the vulnerability to erosion of a watershed. This is further demonstrated on St. John by the construction of Centerline Road in upper portion of the Coral Bay watershed, which was directly accompanied by an increase in terrigenous sedimentation rates in the coast [49].

Fortunately, the AGMD variable is in most cases relatively easy to through public records, satellite imagery, and/or a community-based research effort. The original vector shapefiles used in the AGMD dataset for the high-resolution EVI-STJ were started by a local watershed action group the Coral Bay Community Council (CBCC). After receiving those files, we were able to fill in data gaps via manual identification using satellite imagery, communication with locals about what existed where, and simple, directed GPS surveys using a handheld GPS unit.

It should be noted that this method does not take into account coastal vegetation such as mangroves that trap some sediments before reaching the bay [79]. Future methods will attempt to incorporate coastal vegetation as a critical component of this analysis allowing us to understand how it impacts coastal deposition and potential alterations to critical coastal environments.

## Conclusions

Vulnerability to land-based erosion in small (5,000 km^2^) islands of the tropics is important to consider for land use management but a challenge using large-scale global datasets. We develop a methodology to overcome these limitations using higher-resolution island-specific data tested on St. John in the U.S. Virgin Islands, that yields a more accurate and clearer vulnerability assessment. Some island-specific data are particularly important to have including anthropogenic development (roads and buildings), watershed slope, and earthquake probability. Roads and buildings are particularly important to locally constrain because they are difficult to identify in heavily forested regions using satellite algorithms and the rapid, ongoing rate of development can quickly lead to outdated and underestimations.
